# Identification of new deep sea sinuous channels in the eastern Arabian Sea

**DOI:** 10.1186/s40064-016-2497-6

**Published:** 2016-06-23

**Authors:** Ravi Mishra, D. K. Pandey, Prerna Ramesh, Peter D. Clift

**Affiliations:** IODP-India, National Centre for Antarctic and Ocean Research, Headland Sada, Vasco-Da-Gama, Goa 403804 India; National Centre for Antarctic and Ocean Research, Headland Sada, Vasco-Da-Gama, Goa 403804 India; Department of Geology and Geophysics, Louisiana State University, Baton Rouge, LA 70803 USA

**Keywords:** Submarine canyon, Deep sea channel system, Indus Fan, Arabian Sea

## Abstract

Deep sea channel systems are recognized in most submarine fans worldwide as well as in the geological record. The Indus Fan is the second largest modern submarine fan, having a well-developed active canyon and deep sea channel system. Previous studies from the upper Indus Fan have reported several active channel systems. In the present study, deep sea channel systems were identified within the middle Indus Fan using high resolution multibeam bathymetric data. Prominent morphological features within the survey block include the Raman Seamount and Laxmi Ridge. The origin of the newly discovered channels in the middle fan has been inferred using medium resolution satellite bathymetry data. Interpretation of new data shows that the highly sinuous deep sea channel systems also extend to the east of Laxmi Ridge, as well as to the west of Laxmi Ridge, as previously reported. A decrease in sinuosity southward can be attributed to the morphological constraints imposed by the elevated features. These findings have significance in determining the pathways for active sediment transport systems, as well as their source characterization. The geometry suggests a series of punctuated avulsion events leading to the present array of disconnected channels. Such channels have affected the Laxmi Basin since the Pliocene and are responsible for reworking older fan sediments, resulting in loss of the original erosional signature supplied from the river mouth. This implies that distal fan sediments have experienced significant signal shredding and may not represent the erosion and weathering conditions within the onshore basin at the time of sedimentation.

## Background

Deep sea channel systems are recognized as important components of continental margin bathymetry, due to their pivotal role in shaping the morphology of submarine fans. Submarine fans are the largest clastic accumulations on Earth and receive sediment through canyon-channel systems. The sediment transfer zones between terrestrial sources and deep sea depositional sinks include submarine canyon-channel systems, which generally transition from erosional V-shaped canyons indenting the upper and mid slope of the continental shelf, to U-shaped channels with over bank deposits across the lower continental slope and rise (Covault 2011). Despite this role in connecting the continent and the deep sea it is not always clear how canyons transform from being incisive on the continental slope to being constructive on the abyssal seafloor. If sediment is stored and reworked from locations along a channel system then this may be a source of signal shredding between the source and ultimate sink, in addition to the shredding seen in alluvial flood plains (Castelltort and Van Den Driessche 2003; Jerolmack and Paola 2010). Quantifying the buffering role that channel systems play and how this evolves downslope is best achieved by better mapping of active channels on the largest submarine fans. This is crucial if the abyssal turbidite record is to be used to understand evolving continental environmental conditions in the source regions.

Although the basic model in which avulsing depositional lobes migrate across the surface of a submarine fan has been well-established, it is often unclear what controls the architecture of a channel belt in detail and thus the distribution of the subsequent sedimentary deposits. Examples within the geological record are documented but typically lack the spatial extent to be able to fully understand how the basin geometry controls channel morphology. In foreland basins the longitudinal aspect of the basin usually acts to guide the geometry of the channels (De Ruig and Hubbard 2006) but in more open systems such as deep-sea basins the role of ridges or seamounts is less well-defined.

Since the advent of marine data acquisition techniques in the late twentieth century, high-resolution bathymetry, marine seismic and deep-tow sonar equipments have made it possible to investigate deep water canyon-channel systems (Normark 1970; Damuth et al. 1983). Canyon-channel systems in various submarine fans have been studied in detail by many researchers e.g. Gorsline (1970), Prins et al. (2000), Curray et al. (2003), Bourget et al. (2010), Normark (1970, 1978), Covault et al. (2012), Deptuck et al. (2003), Wynn et al. (2007), and Mishra et al. (2015). The cross sections and transverse profiles of various deep sea channel systems have been studied to understand the channel morphology, geometry, slope and even the channel migration (Griggs and Kulm 1970; Mahar and Zaigham 2013; Bourget et al. 2010; Curray et al. 2003; Kamesh Raju et al. 1993; Subrahmanyam et al. 2008; Babonneau et al. 2002). Though there have been numerous studies of submarine canyons and deep-sea channel systems worldwide, very little has been reported from the Indian Ocean due to lack of data, although some profiles across the Indus Canyon were presented by Kolla and Coumes (1991), McHargue and Webb (1986) and Clift et al. (2014).

The Indus Canyon system, situated in the Arabian Sea (Deptuck et al. 2003; Clift et al. 2014), is the second largest canyon system worldwide after the Bay of Bengal (Bouma et al. 1985). The morphological features of the eastern Arabian Sea have been previously studied using single beam, satellite altimetry and conventional Hydrosweep multibeam systems (Basu et al. 1994; Das et al. 2007; Hillier and Watts 2007; Rao et al. 1992; Bhattacharya and Subrahmanyam 1991; Bhattacharya et al. 1994; Iyer et al. 2012). There are several channels that have been reported by previous workers in the middle and lower Indus Fan and channel systems have been reconstructed (Kenyon et al. 1995; Mackenzie 1997; Prins et al. 2000; Mishra et al. 2015; Prerna et al. 2015). Active channels in the middle Indus Fan have been identified from multibeam bathymetry at 20°N (Fournier et al. 2011; Rodriguez et al. 2011). Geological Long Range Inclined Asdic (GLORIA) side scan sonar (Kenyon et al. 1995; Prins et al. 2000) was used to map channels in the middle Indus Fan, west of the Laxmi Ridge. Sinuous channels have also been mapped by multibeam bathymetry in the lower Indus Fan at 12°–13°N latitude and 67°–68° 30′E longitude (Kodagali and Jauhari 1999).

**Fig. 1:**
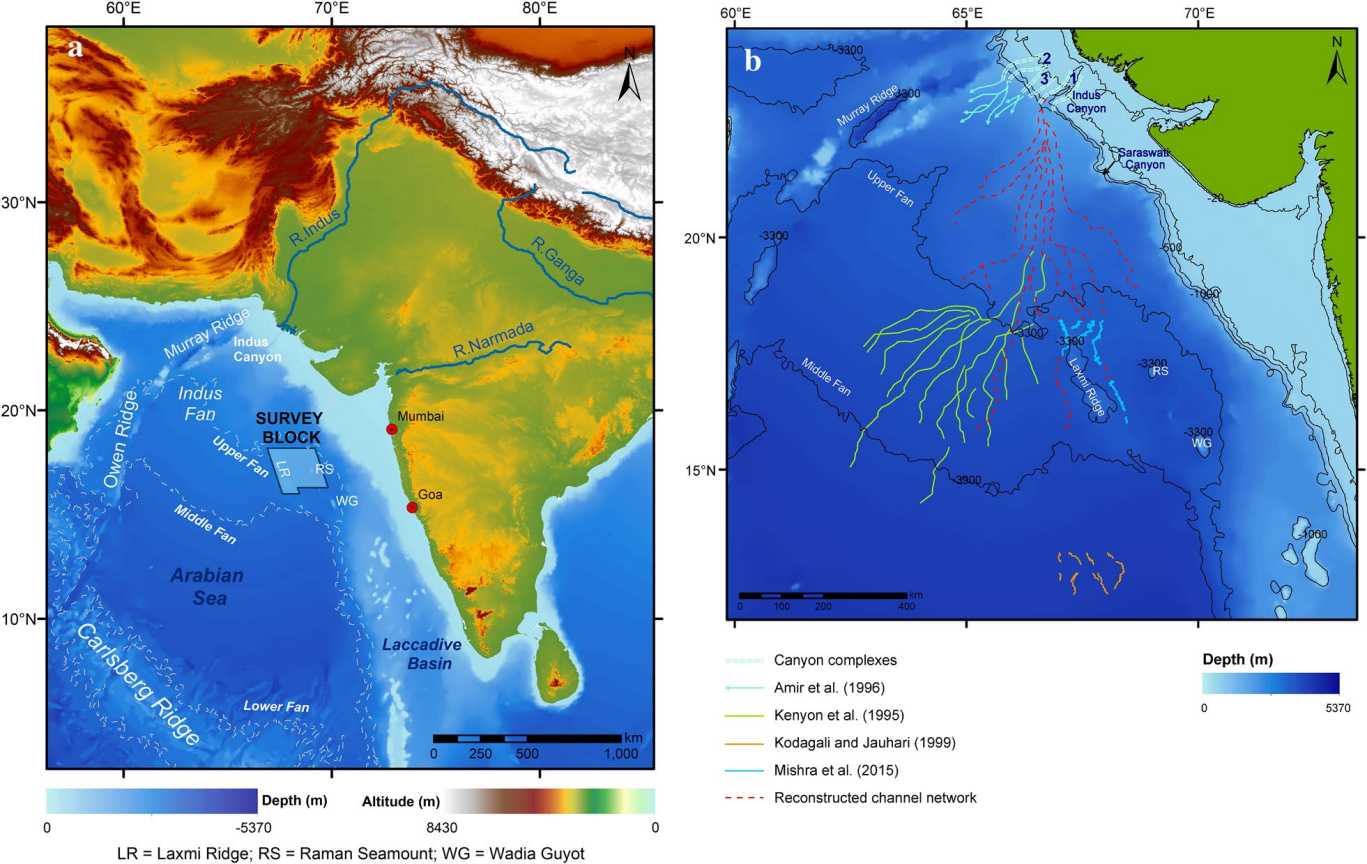
**a** Land-to-deepsea sediment routing system of River Indus flowing from Himalayan region and depositing sediments to the Arabian Sea forming the Indus Fan is depicted here. Indian subcontinent; upper, middle and lower Indus Fan margins; and major fluvial rivers such as Indus, Ganga and Narmada are also shown in the map. Survey block, in which channels discussed in this study, is marked for reference. **b** Reconstruction of Indus channel system from global bathymetry data is marked. Channels identified in upper and middle fan from Kenyon et al. (1995) and Prins et al. (2000); channels from lower fan identified by Kodagali and Jauhari (1999) are marked. Channels identified by Mishra et al. (2015) which are explained in greater detail in this study are also shown. A reconstruction of the channel network from the Indus Canyon to the identified channels using global bathymetric data (Smith and Sandwell 1997) is portrayed for creating a generic pattern of channel network in the upper Indus Fan (Prerna et al. 2015)

**Fig. 2:**
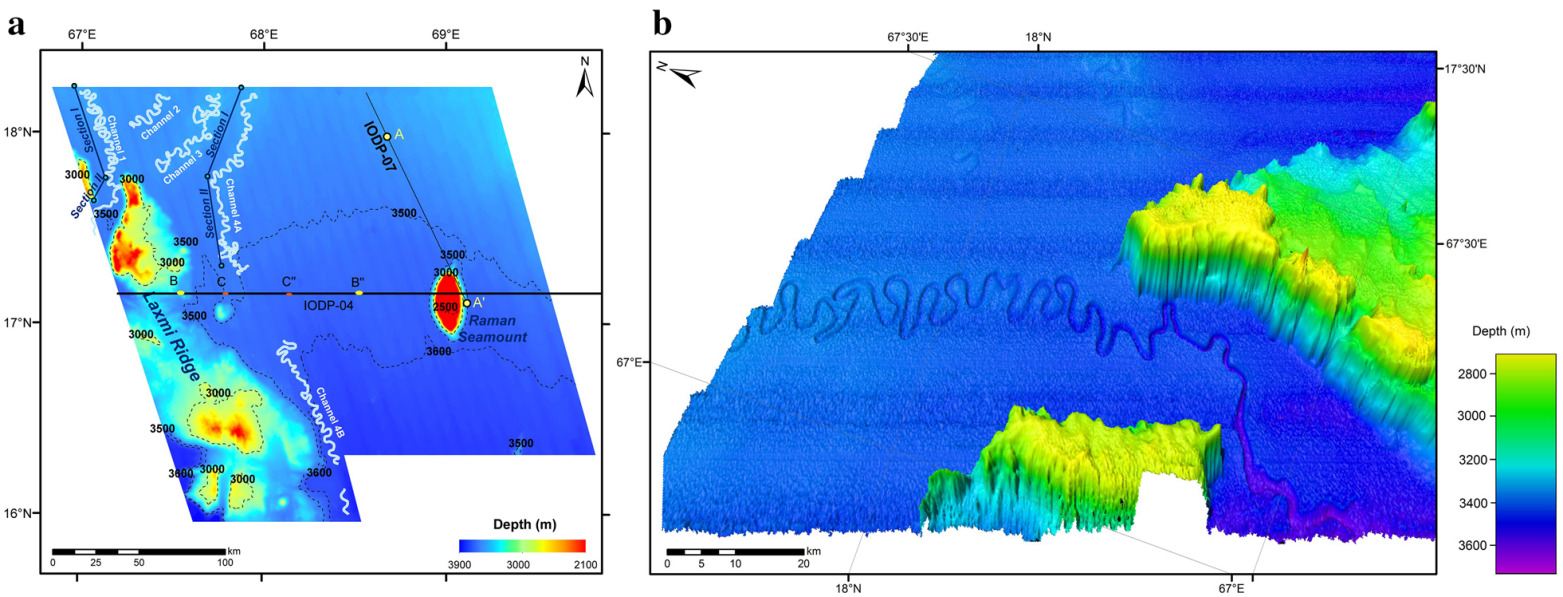
**a** Bathymetry map of the study area as derived from swath bathymetric data acquired using Multibeam SB 3012 system. The ensonified features—Laxmi Ridge, Raman Seamount and submarine channels (1, 2, 3, 4A, 4B) have been marked. Contours represent depth ranging 2100–3900 m BMSL (Below Mean Sea Level) at an interval of 100 m; Seismic tracks IODP-04 and IODP-07 used in this study are also marked. Segments AA′, BB′ and CC′ are elaborated in Figs. 3c, 4 and 5 respectively. **b** 3D visualization of Channel 1 flowing in between the northern reaches of Laxmi Ridge is provided here. Vantage point is WSW–ENE. Vertical exaggeration is increased to 5× in order to highlight the south-ward deepening trend followed by Channel 1. Channel 2 and 3 can also be lightly identified towards NE of Channel 1

The channels identified in the present study (Fig. 1) are part of middle Indus Fan and their connectivity has been established with the main Indus Canyon-channel system (Prerna et al. 2015), using satellite gravity derived bathymetry data (Smith and Sandwell 1997). This paper presents more detailed study of Indus Fan system based on the interpretation of new bathymetry data. We have described the morphology and structure of the active channel systems and also identified new channels proximal to the eastern flanks of the Laxmi Ridge in the Arabian Sea (Fig. 2). The Indus channel system has been reconstructed based on the new identified channels (Fig. 1b).

## Regional setting

The Arabian Sea, located in north-western Indian Ocean, contains the Indus Fan which is the dominant sedimentary feature in the region (Fig. 1). The Indus Fan is located at the junction between the Arabian, Eurasian and Indian Plates and is juxtaposed against the Western Continental Margin of India (WCMI), which is passive continental margin (Fig. 1a). At the north-western periphery of the fan, the Eurasian plate over thrusts the Arabian plate forming the Makran Accretionary Prism (McCall 1997). The transform boundary between the Arabian and Indian plates extends from the Owen Ridge (Owen Fracture Zone, Fig. 1), north to the Murray Ridge and onshore to the Chaman Fault (Fournier et al. 2008). The Carlsberg Ridge is located along the southwest periphery of the fan, whereas in the east it is bounded by the marginal highs associated with the WCMI (Fig. 1a).

The WCMI evolved after Gondwana break-up (about 90 Ma) and is about 40 m.y. younger than the Eastern Continental Margin of India (ECMI) (Subrahmanyam and Chand 2006). Subsequent rifting and seafloor spreading during the middle Cretaceous (India–Madagascar break-up) as well as India–Seychelles break-up during late Cretaceous (MacKenzie and Sclater 1971; Naini and Talwani 1982; Minshull et al. 2008) gave rise to the WCMI. Sediment accumulation began to speed up along this passive margin following the late Oligocene to early Miocene (Clift et al. 2001), India-Eurasia collision and initial Himalayan uplift during the early Eocene (Najman et al. 2010; Sahni and Kumar 1974; Dewey and Bird 1970). The post-rift Himalayan fan sedimentation is believed to have been underlain by the earlier pre and syn-rift deposits derived from peninsular India in this region (Clift et al. 2002; Pandey and Pandey 2016).

### Terrestrial to marine sediment routing system of River Indus

#### River Indus and delta

The Indus River is one of the major rivers of Asia, which originates on the Tibetan Plateau and flows south-westerly through alluvial plains traversing around 3200 km before reaching its delta (Fig. 1a) at the Arabian Sea (Mirza 2005; Inam et al. 2007). The terrestrial Indus basin covers approximately 1.12 million km^2^ (Hartmann and Andresky 2013). The Indus River drains barren, unconsolidated glacial and fluvial reworked detritus eroded from high-relief, rapidly uplifting tectonic units of the western Tibetan Plateau, Karakoram and Himalaya (Milliman et al. 1984; Clift 2002); this supplied to the fifth largest river sediment load in the world prior to damming in the last century (Wells and Coleman 1984).

Sediments are either deposited in the delta, which covers 8000 km^2^ (Clift and Giosan 2014), or bypass through the Indus Canyon from where they may subsequently be deposited into the deep sea abyssal plain (Kolla and Coumes 1984; Prins et al. 2000).

#### Indus Canyon and channel system

Canyon-channel systems provide routes to transport and deposit sediment into the deep-sea and are primarily observed along continental margins. Deepwater canyon-channel systems have been identified in various geographic contexts, but their seaward extent is limited to a few hundred kilometers from the shelf and only some canyons extend beyond 1000 km (Covault et al. 2012).

The two largest canyon-channel systems of the world are in the Indian Ocean, namely the Ganga–Brahmaputra system in Bay of Bengal and the Indus system of Arabian Sea. The prominent Indus Canyon and its associated deep-sea channel system can be observed from multibeam bathymetry and satellite derived global bathymetry data (Von Rad and Tahir 1997; Ryan et al. 2009; Clift et al. 2014). The Indus Canyon, classified as a delta front trough (Shepard and Dill 1966), creates an indent on the ~100 km wide continental shelf. The canyon extends across the continental slope with an average width of 8 km and a maximum depth of 1200 m at the shelf edge. At 1400 m water depth, the canyon widens to 20 km and is 325 m deep (Wynn et al. 2007 and references therein). In that area the canyon transitions into the depositional channel levee systems on the upper fan. Erosional channels extending on to the middle fan are smaller, with depth ranging 30–40 m and relatively small levees, while the lower fan is characterized by numerous small channels (<1.5 km wide and <5 m deep) with or without small levees (Wynn et al. 2007).

Only one canyon-channel system has been active on the Indus margin at any one time (Bourget et al. 2013; McHargue and Webb 1986; Kolla and Coumes 1987; Kenyon et al. 1995; Von Rad and Tahir 1997; Prins et al. 2000; Wynn et al. 2007). The modern active Indus channel-levee system has been mapped by GLORIA long range side scan sonar (Kenyon et al. 1995; Prins et al. 2000) and the distal part of this system was further imaged with a multibeam swath bathymetry system (Kodagali and Jauhari 1999; Mishra et al. 2015). At present, the active Indus Canyon (185 km long and up to 1.6 km deep) is the main feeder canyon (Fig. 1b) of the Indus submarine fan (Kenyon et al. 1995; Von Rad and Tahir 1997; Prins et al. 2000; Bourget et al. 2013; Clift et al. 2014).

Three palaeo-canyon systems have been recognized in the shelf and upper slope area of the Indus Fan (Fig. 1b), two of which lie to the west of the modern active Indus Canyon, while a third (the Saraswati Canyon) is to the southeast (McHargue and Webb 1986; Kolla and Coumes 1987; Droz and Bellaiche 1991; Clift et al. 2002; Deptuck et al. 2003; Bourget et al. 2013). In the presently active Indus Canyon system, temporal changes over the last ~100 years have included westward canyon shifting (Mahar and Zaigham 2013).

#### Indus Fan

As turbidity currents reach abyssal plains, a decrease in slope causes reduced velocity, allowing sediments to be deposited within lobes. Construction of the fan is primarily due to the deposition of sediments transported by turbidity currents (Naini and Kolla 1982; Kolla and Coumes 1984). The Indus Fan extends about 1500 km into the Arabian Sea and is up to 960 km wide (Naini and Kolla 1982; Kolla and Coumes 1984, 1987).

## Datasets

High resolution swath bathymetry data were acquired onboard ORV Sagar Kanya (SK-306) in 2013 in the eastern Arabian Sea. 2D multi-channel seismic data were collected along four regional lines within the survey block in 2014. Processing and interpretation of this high resolution multibeam and multi-channel seismic data forms the basis of this study. Swath bathymetry data from the eastern Arabian Sea were acquired onboard ORV Sagar Kanya, during a 26 day long cruise SK-306, using a hull-mounted deepwater SB3012 multibeam system. The swept-beam technology provides full motion compensation and the system operates at 12 kHz frequency with an effective 150° of swath and a beam width of 1°, providing coverage over five times the water depth. A total of around 7740 line km data were collected along 27 track lines, covering an area of 54,253 km^2^. The multibeam system uses Hydrostar software for data acquisition and C-NAV, dual frequency, DGPS system is used for positioning. Data were processed with EIVA©Nav Editor Software and subsequently exported into ArcGIS v10.2 for creating 2D Digital Bathymetric Model(s) of the survey area and into Fledermaus v7.3.1a for generation of 3D plots. In addition, supporting 2D seismic data were acquired under the International Ocean Discovery Program of India (IODP-India). 2D seismic data of 12 s Two Way Travel Time (TWT) were acquired along four profiles onboard R/V Geo Hindsagar, with a gun volume of 5500 cu in. at 2000 psi and 6000 m long streamer. The location of the study area within the Indus Fan system is shown in Fig. 1a. Seismic data were processed up to time migrated stacks using a standard approach prior to interpretation.

## Results

### Morphology

**Fig. 3:**
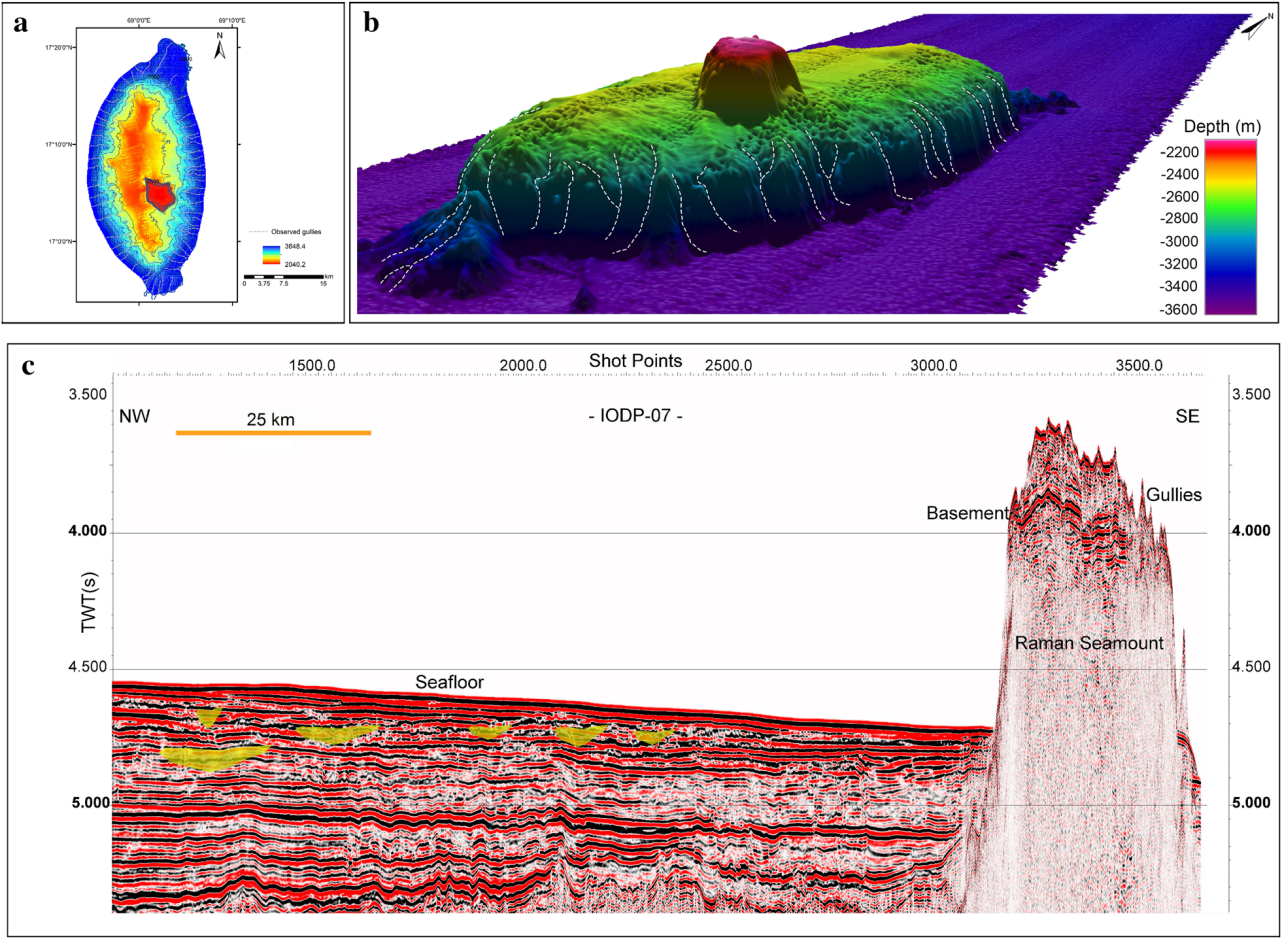
**a** 2D image of Raman Seamount with dendritic gully pattern. **b** 3D image of Raman Seamount with a vantage point of SE–NW. Secondary peak above the plateau of Raman Seamount is depicted. Gully patterns can be seen on the flanks of the feature and marked as dashed lines. **c** 2D seismic reflection profile (segment AA′ from IODP-07; (refer Fig. 2) showing Raman Seamount and presence of various paleo-channels

A bathymetry map of 100 m grid size (Fig. 2a) and three dimensional images (Fig. 2b) were generated from SB3012 multibeam data. The overall bathymetry is rather gradual with water depths varying from 3000 to 3700 m. The most prominent morphological features are the Laxmi Ridge and Raman Seamount (Figs. 2, 3). The seismic reflection and the multi-beam bathymetry data employed in this study image the Raman Seamount in detail for the first time. The Raman Seamount is a part of NNW-trending linear seamount chain consisting of three major edifices, Raman and Panikkar Seamounts and Wadia Guyot (Bhattacharya et al. 1994). Laxmi Ridge is a NW–SE elongated structure considered to be a continental sliver rifted from India before the Seychelles–India break-up (Naini and Talwani 1982; Biswas 1987; Kolla and Coumes 1990; Talwani and Reif 1998).

The multibeam data presented in this study gives enhanced three-dimensional perspectives of the channel systems in the distal parts of the fan and further allows precise images of the Raman Seamount and Laxmi Ridge. Laxmi Ridge, is ~246 km long, 86.5 km wide and on average 835 m high, covering an area of about 10,640 km^2^ (Table 1). The N–S elliptical and elongated morphological expression-Raman Seamount is about 49 km long and 23 km wide, with an average height of 1371 m, covering a basal area of around 772 km^2^ (Table 1). Earlier studies reported a secondary peak on the top of the Raman Seamount having basal area of about 28 km^2^ (Bhattacharya et al. 1994; Iyer et al. 2012). The secondary peak and its basal area (34 km^2^) measured based on new results are in conformity with the earliest studies (Fig. 3a, b).

**Table 1:** Dimension of the Raman Seamount and Laxmi Ridge based on new data

Parameters	Raman Seamount	Laxmi Ridge
Basal area (km^2^)	772.07	10,640.13
Basal area of secondary peak (km^2^)	34.36	–
Plateau area (km^2^)	208.15	–
Maximum height (m)	1371	835
Length (km)	48.94	246.06
Width (km)	22.86	86.43

### Fan sedimentation

**Fig. 4:**
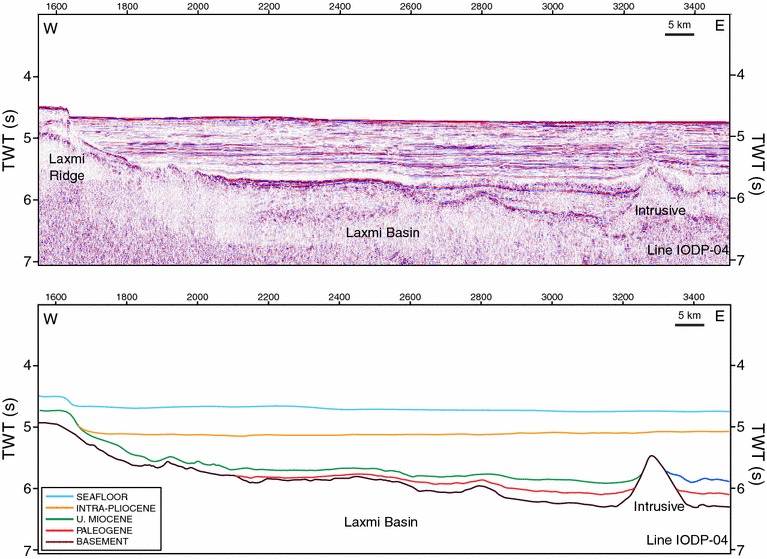
Profile of seismic track IODP-04 (Section AA′ refer Fig. 2) and interpreted (*bottom*) seismic section. *TWT*(*s*) two-way travel time in seconds

**Fig. 5:**
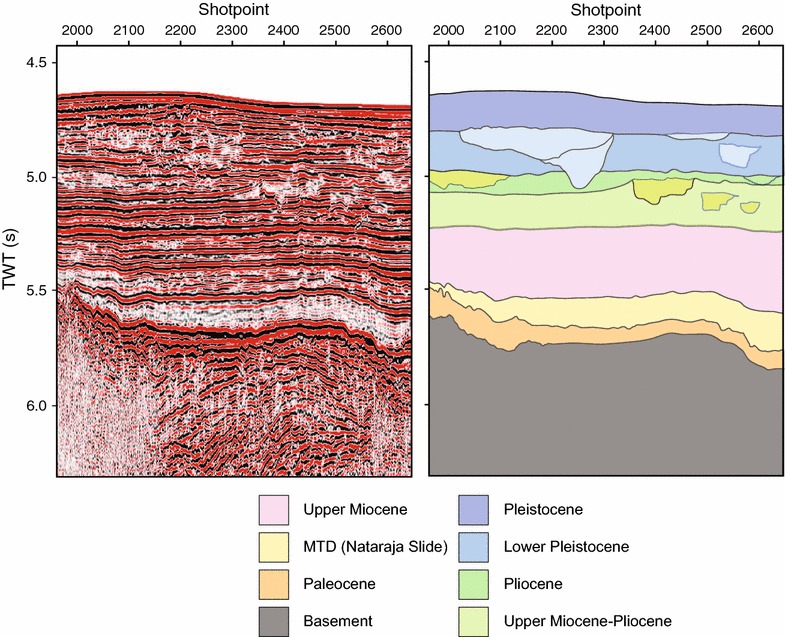
Close-up (*left*) and interpretation (*right*) of seismic profile IODP-04 (Section CC′) showing the major seismic units identified in IODP-355 drilling. The sections show large channels in the Pleistocene section. *MTD* mass transport deposit. The *different colours* represent resolvable seismic units

Seismic profiles from the area have been described by Pandey et al. (2015). Seismic profile IODP-04 (Figs. 4, 5) demonstrates the presence of thick upper Miocene and younger strata (~460 m). The fan in the area of the Laxmi Basin is built up by a series of overlapping distal lobes that are cut by erosive channels, presumably supplying lobes in yet more distal parts of the fan. The lobes do not show well developed aggradation channel levee forms, as better developed in the western and northern Arabian Sea. In those more proximal settings the characteristic triangular cross section of an aggradation channel is clear on seismic (McHargue and Webb 1986; Droz and Bellaiche 1991; Clift et al. 2002) contrasting with the flatter geometries seen in this area. The channels in the sub-surface of the Laxmi Basin moreover incise into the older strata and are often not aggradational, indicating that currents here are less laden with sediments than in more proximal areas. The fact that they are reworking the older strata would result in a recycled sedimentary record downslope that only partly reflects the erosional signal from the source at the time of sedimentation.

### Deep-sea channel systems

**Fig. 6:**
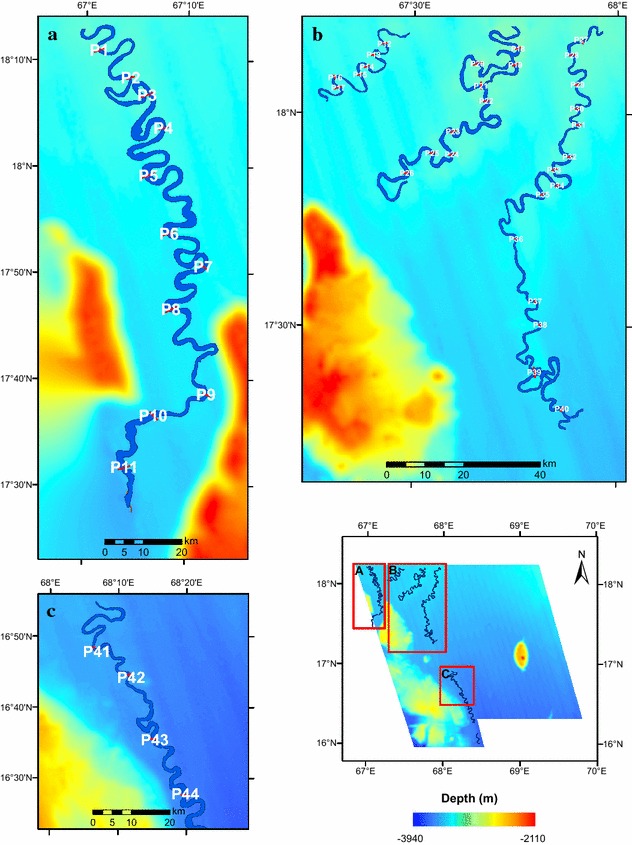
The location of 2D depth profiles for Channels 1 to 4B (refer Fig. 7 and Appendix: Figs. 8, 9, 10, 11) is presented. **a** Profiles P1 to P11 (Channel 1); **b** profile P12 to P40 (Channel 2, 3, 4A); **c** profile P41 to P44 (Channel 4B). *Inset* map of study area showcases the extent of Blocks A to C

A total of five channels have been observed in the area and are shown in Figs. 2 and 6. Channel 2, 3 and section I of Channel 4A (NE of Laxmi Ridge) have a NE–SW orientation, perpendicular to the ridge. The orientations of Channel 1, section II of Channel 4A and Channel 4B follow a track parallel to the Laxmi Ridge. Channel 4B could be connected with Channel 4A. We divide Channels 1 and 4A into two sub-sections (I and II) based on a sudden change in orientation. A 3D model of a part of Channel 1 is shown in Fig. 2b. Channel dimensions are computed and listed in Table 2. The total length of all channels along the channel axis is about 915 km, and their width varies from 189 to 1980 m. Channels have an average depth of about 60 m. The longest, Channel 4A, is about 256 km long, 700 m wide and about 57 m deep (Table 2).

**Table 2:** Dimension of the Indus channel system in the area

Indus channel system	Channel 1			Channel 2	Channel 3	Channel 4A			Channel 4B
	Total	Section I	Section II			Total	Section I	Section II	
Channel axis length (km)	226.06	171.42	54.63	69.54	179.36	256.30	121.31	120.49	183.62
Downstream straight distance (km)	89.42	54.14	35.28	27.67	55.81	112.30	55.69	56.61	79.04
Sinuosity	2.53	3.17	1.55	2.51	3.21	2.28	2.18	2.12	2.32
Slope gradient of channel	1:511	1:694	1:368	1:864	1:649	1:702	1:831	1:1029	1:564
Width range (m)	352.67–1873.49	352.67–1873.49	418.92–1775.93	189.81–657.84	460.71–1290.95	274.67–1147.36	274.67–939.65	526.84–1174.36	412.54–1980.49
Depth range (m)	19.66–95.33	26.37–90.62	19.66–95.33	9.33–28.66	36.55–71.44	20.66–82.8	20.66–58.33	25.2–82.8	7.5–91.5
Avg. depth of channel (m)	66.42	61.85	70.99	52.16	55.69	57.45	60.51	54.44	58.68

### Channel morphology and sinuosity

**Fig. 7:**
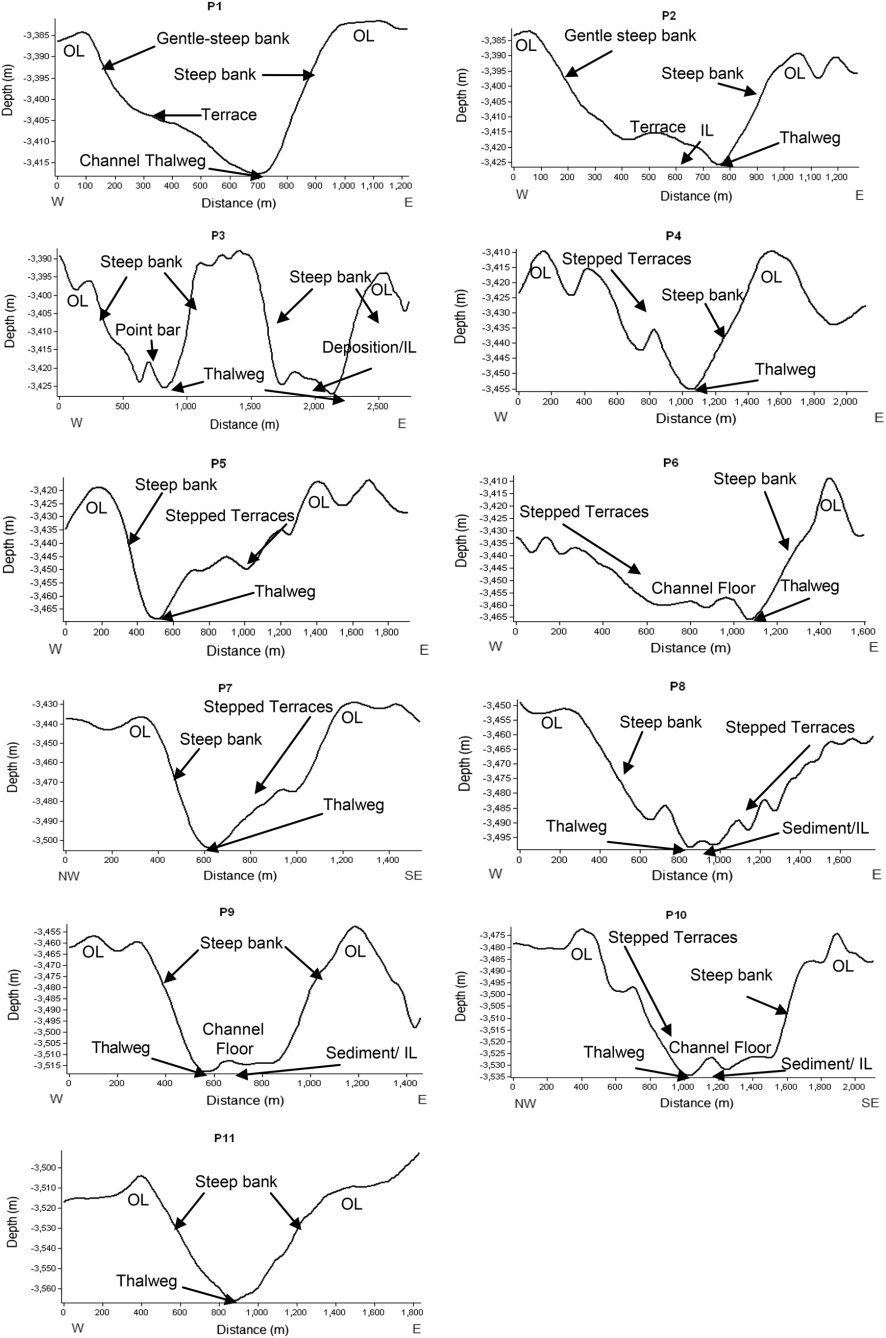
Profiles P1 to P11 showing the morphological behaviour of Channel 1. The channel notably gains gradient from north to south as can be observed from the profile views. Typical depositional features like stepped terraces, levees and point bars are clearly visible. (*OL* outer levees, *IL* inner levees)

Cross section profiles were generated to cover the entire channel system (Fig. 6) from the gridded depth data and are shown in Fig. 7 and the “Appendix”. A total of 44 profiles are shown, which include eleven profiles from Channel 1, six from Channel 2, nine from Channel 3, fourteen from Channel 4A and four profiles from Channel 4B. The profiles show the channel thalweg, as well as outer levees, channel banks, terraces, stepped terraces, and inner levees.

Multi-channel seismic (MCS) reflection data have been used to understand the seafloor. The presence of the channels (or the lack thereof) in proximity to major features such as Laxmi Ridge and Raman Seamount is inferred based on apparent incisions in the seismic sections and comparison with the bathymetry maps. Details of gullies (Fig. 3a, b) over the Raman Seamount identified from bathymetric data are well supported by the seismic images (Fig. 3c).

The sinuosity is a function of slope and nature of sediment, with high sinuosity associated with low gradient. The channels are highly sinuous in nature and average channel length is twice the straight channel length. Channel sinuosity varies from 1.55 (Section II of Channel 1) to 3.21 (Channel 3).

## Reconstruction of the Indus Fan channel system

Channels of the Indus Fan have already been reconstructed west of Laxmi Ridge (Kenyon et al. 1995; Prins et al. 2000), while we now show the presence of channels to the east of Laxmi Ridge. New channels identified are traced back and connected to the upper fan system using the previous data, as well as satellite gravity derived bathymetry data (Fig. 1b). Channel patterns on the Indus Fan are discussed in detailed by Prerna et al. (2015).

The channels imaged here are part of the Indus system and may not be connected with other Indian River systems emanating from the WCMI. Many rivers like the Narmada and Tapti flow into the Arabian Sea from the peninsular but no active canyon-channel system has yet been identified. This contrasts with the western Arabian Sea (NW of Murray Ridge), where many small active canyon-channel systems have been observed (Bourget et al. 2011). The reason for the lack of flow from peninsular India may be attributed to the extremely broad shelf on the western continental margin with comparatively low sediment flux from these rivers causing the lack of canyon-channel system development on the slope, although these rivers are reported to have some offshore extension (Vora et al. 1991). The active channel system of the area mapped in this study shows the eastward extension of the Indus channel system into the middle fan area, and confirms the sediment supply through the shelf region to middle fan area (Fig. 1b).

## Discussion

Indus submarine channel is a sinuous channel system similar to many other modern submarine systems, such as the Amazon, Bengal and Zaire (Clark et al. 1992; Peakall et al. 2012; Wynn et al. 2007). The variations in channel morphology from north to south are notably influenced by the Laxmi Ridge. The channels north of Laxmi Ridge are more sinuous than those to the east of the ridge (Figs. 2, 6; section II of Channel 1 and 4A and Channel 4B). The east–west slope of Laxmi Ridge influences the flow by guiding channels along the ridge trend, i.e. flowing parallel to the ridge as they approach it. The east–west slope of the ridge also restricts expansion of meanders and consequently the channels have comparatively low radii of curvature. These variations on the flow pattern can be attributed to the morphological dominance of the Laxmi Ridge. In this respect these channels mimic those observed in the Austrian Molasse basin in which the topography on the edge of the basin plays a strong role in guiding the orientation of the channels and in turn controlling long-term distribution of sediment types (Hubbard et al. 2009). The similar impact are also observed in African Angola basin (Gee and Gawthorpe 2006). The guiding influence of Laxmi Ridge is also apparent over long geological time periods as revealed by IODP drilling that shows a preferential development of coarser sediments in the basin center and finer lithologies more associated with distal overbank deposits closer to the edge of Laxmi Ridge itself (Pandey et al. 2015).

Channels 4A and 4B were likely connected in the recent past and it seems likely that several of the channels present state represents avulsion events. Channel 2 likely formerly connected to Channel 1, while Channel 3 ends against the edge of Laxmi Ridge and must have been diverted south in the past potentially connecting with Channel 4B before the initiation of Channel 4A. The Indus channels show similarities with punctuated channel migration history of the Lucia Chica Channel offshore California in which channel migration was not continuous but occurred in a number of discrete steps (Maier et al. 2012; Fildani et al. 2013).

The steepest gradient in this study is observed in the channels flowing in the vicinity of the Laxmi Ridge (Fig. 6; section II of Channels 1 and 4A) with comparatively low degrees of meandering. The steep slope at the foot of Laxmi Ridge and its adjoining area, along with the slope gradient of the channel together control channel flow and the meandering in the vicinity of the ridge. The morphology of the channel banks is characterized by steep to gentle slopes, while the inner banks are frequently marked by the presence of one or several small terraces (Fig. 7 and “Appendix”).

Channel morphology and dimensions typically exhibit the channel characteristics of the middle Indus Fan, as described by Kolla and Coumes (1987) and Wynn et al. (2007). In general the maximum width of the channels is present in the upper fan area and because the gradient decreases southward, the channel widths and depths also decrease towards the lower fan. Channels were previously reported to be 10 km wide in the upper fan area, whereas in the lower fan, channels are <1.5 km wide and <5 m deep (Wynn et al. 2007). In all systems, channel widths and depths decrease downstream (Normark 1985), which is also true for the channels mapped here.

The Raman Seamount shows gullies and a secondary peak (Fig. 3). The flat surfaces appears to have been formed either by erosional wave planation effects or by constructional volcanic processes (Fornari and Ryan 1984; Bhattacharya et al. 1994), while extensive gully features are likely the product of submarine erosional processes (Kenyon et al. 1978; Lonsdale et al. 1972). If the Raman Seamount is a similar age of the rest of Laxmi Basin then simple oceanic thermal subsidence of ~2800 m since 65 Ma (Parsons and Sclater 1977) would place the top of the seamount (now at ~2100 m water depth; Fig. 3) above sea level at the time of its initial eruption. Thus wave cutting would be a possible explanation for the remarkably flat top of the seamount.

The dendritic gully pattern over the seamount resembles a relict drainage pattern of subaerial erosional origin but these gullies must be submarine because drilling of the basement at IODP Sites U1456 and U1457 adjacent to Laxmi Ridge and Raman Seamount indicates consistent deep-water conditions that preclude subaerial emergence of the seamount in recent times (Pandey et al. 2015). Similar systems have been noted in other continental margin settings, such as California (Pratson and Ryan 1996), Central Atlantic America (Vachtman et al. 2013), Chile (Bernhardt et al. 2015) and Taiwan (Ramsey et al. 2006), where the channels are all clearly formed by submarine processes alone. Vachtman et al. (2013) hypothesize that a concave hypsometry is characteristic of erosion by sedimentary flows, spilling over the shelf edge. In contrast a convex hypsometry that dendritic networks, originating at or below the shelf break, as we see in this study, is attributed to retrograde erosion as a result of headward erosion driven by mass wasting.

The youngest sediments in Laxmi Basin are muddy and carbonate rich, whereas older sequences are sandy sheet-like deposits. The initial results at IODP Sites U146 and U1457 indicate the top ~70 mts < 1.2 Ma, spanning much of the Pleistocene (Pandey et al. 2015), during which time there have been many sea-level cycles and plenty of opportunities for the accumulation of “low stand” fan deposits. Since 1.2 Ma the active lobes of the Indus Fan have been located west of Laxmi Ridge, allowing a dominantly muddy and hemipelagic sequence to accumulate (Pandey et al. 2015).

## Conclusions

This study presents a detailed mapping of new channel systems in the eastern middle Indus Fan and for the first time highlights the pathways of Indus Canyon-channel system, on the eastern side of Laxmi Ridge. The morphometric analyses confirm that the channels observed are fed by the currently active Indus Canyon-channel complex system and become less sinuous going south as they are guided in their trajectory by the mass of the Laxmi Ridge. The channels are inferred to be in the mature stage based on their high sinuosity. The origin of the newly discovered channels has been traced back through the middle and upper Indus Fan channel systems using flow accumulation and flow direction modelling in conjunction with the regional bathymetry data. They are not all connected and are likely to be the products of punctuated avulsion events. They are not fed by rivers flowing from western India and are most likely the modern equivalents of erosive channels seen in the Pliocene-recent sub-surface. These channels have the capacity to recycle significant amounts of sediment and may contribute to the loss of erosional signal between the river mouth and the deep abyssal seafloor. The presence of the active channels beyond 68°E indicates an eastward extension of the Indus Canyon-channel system. Bathymetric mapping has further imaged the Raman Seamount, which is a flat topped feature with a prominent secondary peak. Simple subsidence estimates indicate the flat top might be linked to wave-cutting shortly after emplacement. However, dendritic channels around the edge of the seamount likely reflect the influences of mass wasting and headward erosion in a purely marine setting.

## Figures and Tables

**Fig. 8:**
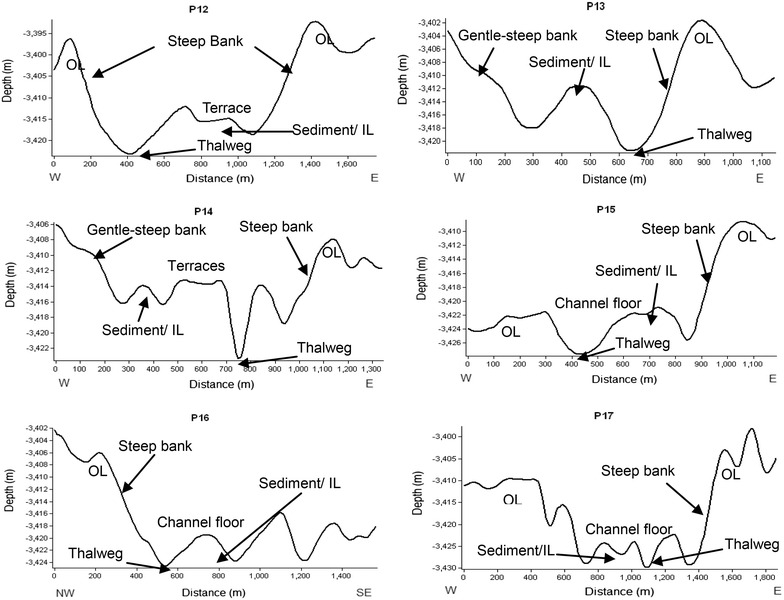
Profiles P12 to P17 showing the morphological behaviour of Channel 2. This channel shows marked variability in its internal structure and follows a NE–SW trend. Typical depositional features like stepped terraces, levees and point bars are clearly visible (*OL* outer levees, *IL* *inner levees*)

**Fig. 9:**
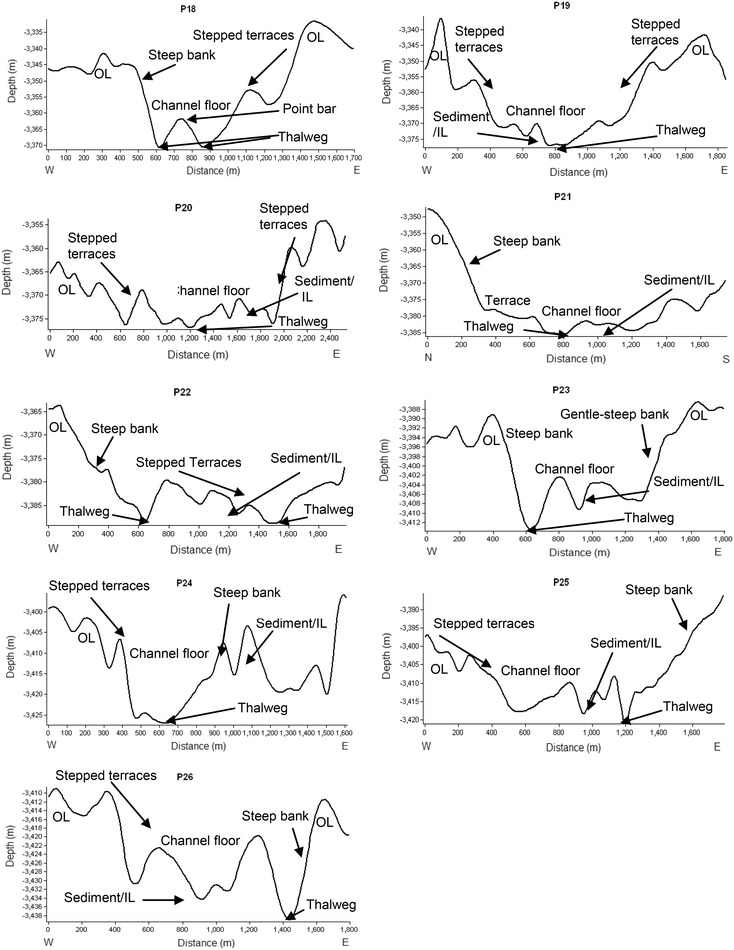
Profiles P18 to P26 showing the morphological behaviour of Channel 3. This channel follows a broader width as opposed to other channels, also following a NE–SW trend. Typical depositional features like stepped terraces, levees and point bars are clearly visible. (*OL* outer levees, *IL* inner levees)

**Fig. 10:**
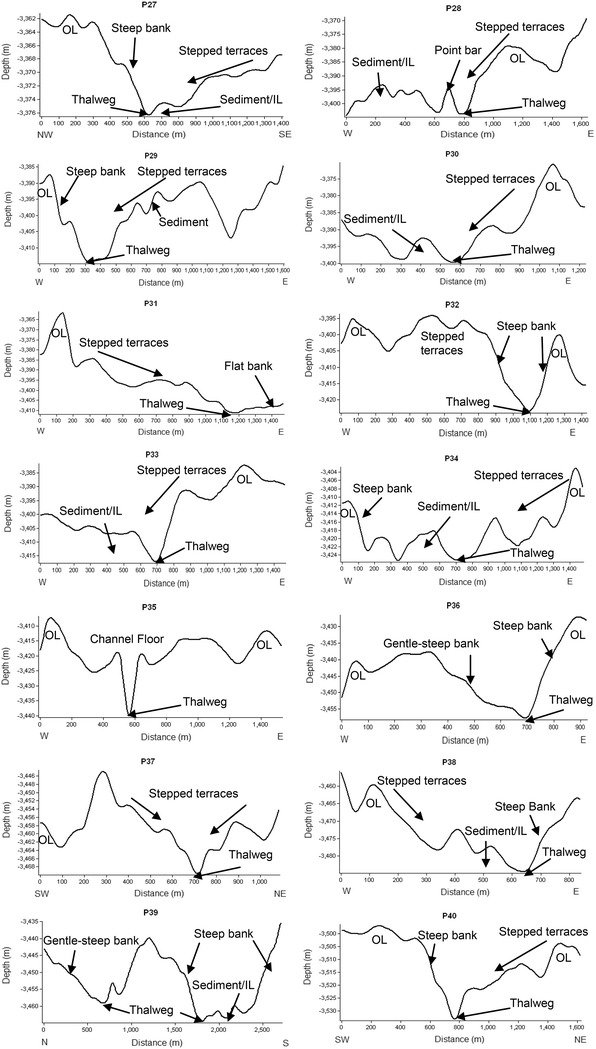
Profiles P27 to P40 showing the morphological behaviour of Channel 4A. Initially flowing from NW–SE, the channel shifts near P36 to follow a more NNW–SSE trend. A higher gradient, similar to the behaviour of Channel 1 can be noted. Typical depositional features like stepped terraces, levees and point bars are clearly visible. (*OL* outer levees; *IL* inner levees)

**Fig. 11:**
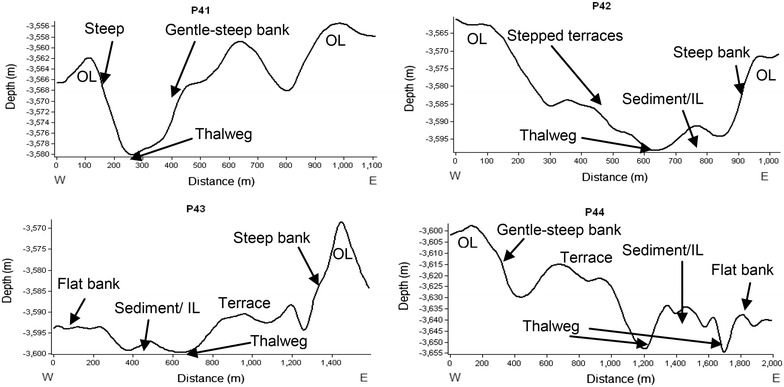
Profiles P41 to P44 showing the morphological behaviour of Channel 4B. Flowing in a clear NNW-SSE trend, it can be said that this channel is a continuation of Channel 4A. However, due to non-identification of channel in between the two, they have been treated separately for this study. Typical depositional features like stepped terraces, levees and point bars are clearly visible. (*OL* outer levees; *IL* inner levees)

## Appendix

See Figs. 8, 9, 10, 11.
